# Investigation of Biodegradation, Artificial Aging and Antibacterial Properties of Poly(Butylene Succinate) Biocomposites with Onion Peels and Wheat Bran

**DOI:** 10.3390/ma18020293

**Published:** 2025-01-10

**Authors:** Emil Sasimowski, Marta Grochowicz, Katarzyna Janczak, Aleksandra Nurzyńska, Anna Belcarz-Romaniuk

**Affiliations:** 1 Department of Technology and Polymer Processing, Faculty of Mechanical Engineering, Lublin University of Technology, Nadbystrzycka 36 Street, 20-618 Lublin, Poland; 2 Department of Polymer Chemistry, Institute of Chemical Sciences, Faculty of Chemistry, Maria Curie-Sklodowska University, Gliniana 33 Street, 20-614 Lublin, Poland; mgrochowicz@umcs.pl; 3 Łukasiewicz Research Network—Institute of Engineering of Polymer Materials and Dyes, Sklodowska-Curie 55 Street, 87-100 Toruń, Poland; katarzyna-klosowska1@wp.pl; 4 Chair and Department of Biochemistry and Biotechnology, Medical University of Lublin, Chodzki 1 Street, 20-093 Lublin, Poland; aleksandra.nurzynska@umlub.pl (A.N.); anna.belcarz-romaniuk@umlub.pl (A.B.-R.)

**Keywords:** composting, artificial aging, biofiller, PBS, antibacterial properties, thermal properties, agro-waste materials, agro-flour filler

## Abstract

The present article focuses on the characterization of the new biocomposites of poly(butylene succinate) (PBS) with fillers of plant origin such as onion peels (OP) and durum wheat bran WB (*Triricum durum*) subjected to composting and artificial aging. The susceptibility to fungal growth, cytotoxicity and antibacterial properties were also examined. The biodegradation of the samples was investigated under normalized conditions simulating an intensive aerobic composting process. It was shown that the tested natural fillers significantly accelerate the biodegradation process of the composition (after 90 days mass loss of PBS 7%) and that the samples with WB degrade much faster (corresponding mass loss 86%) than those containing OP (corresponding mass loss 21%). The remains of the samples after composting were subjected to chemical structure analysis (FTIR), and their thermal properties were determined using differential scanning calorimetry (DSC). It was shown that the degree of crystallinity of PBS and composites increased with the increasing time of composting. In the case of pure PBS, this increase was a maximum of 31.5%, for biocomposite with OP 31.1% and for those containing WB 21.2%. FTIR results showed that cleavage of polymer chains by hydrolysis took place during composting. The tested samples were also subjected to artificial aging under conditions simulating solar radiation and were sprayed with water. After artificial aging, the significant changes in the color of the samples as well as the porosity of their surface was noted, which was mainly due to the effect of photodegradation of both the used OP and WB fillers. Additionally, FTIR analysis indicated that samples were degraded by photooxidation processes. The ability of fungi to grow on the surface of the samples was also tested. The results demonstrate the possibility of using the developed biocomposite materials as a carbon source for the growth of fungi. The antibacterial tests showed that samples containing OP exhibited strong antibacterial properties regardless of their wt.% content. Additionally, a cytotoxicity test was performed on a BJ cell line, demonstrating that none of the tested biocomposites were cytotoxic. Moreover, those with the addition of WB statistically significantly supported the viability of both fibroblast and bacteria cells, showing their biological safety but lack of antibacterial activity.

## 1. Introduction

Commonly used plastics such as polyethylene, polystyrene, polyvinyl chloride, polypropylene and polyethylene terephthalate are not biodegradable. These plastics can be recycled, but only a fraction of them is. In the European Union, the total amount of plastic waste generated in 2021 was 16.13 Mt, of which only about 6.56 Mt was recycled [1]. Globally, 360 Mt of plastic waste was generated in 2020, of which 81 Mt was not disposed of in an environmentally friendly way [2]. A significant part of it ended up in the natural environment, where it threatens various ecosystems. In 2019 alone, about 22 Mt ended up in soils, rivers and oceans, and the leakage of plastic into the environment is expected to double by 2060 [1]. OECD forecasts indicate that by 2040, the amount of accumulated plastic in rivers and oceans alone will increase to 300 Mt, compared to the estimated 152 Mt in 2020 [2]. One way to reduce this unfavorable phenomenon is to replace non-biodegradable plastics with biodegradable and compostable plastics. Therefore, biodegradable plastics and their composites with fillers of natural origin are the subject of numerous studies reported, among others, in review papers [3,4,5,6,7,8,9].

One of the biodegradable materials that arouses great interest among representatives of industry and scientists is poly(butylene succinate) (PBS). This is due to its good properties, such as impact and tensile strength, thermal and chemical resistance, high flexibility as well as very good processability, and especially compostability and biodegradability [10], also in the marine environment [11,12]. PBS has better thermal properties, melt processability and chemical resistance than other aliphatic polyesters. These properties allow for a wide range of applications, including biomedicine, tissue engineering and bone tissue, food packaging, nets and mulching films in agriculture, tableware as well as yarn in the textile industry [13]. Unfortunately, the costs of producing PBS, which is most often made from succinic acid (SA) and 1,4-butanediol (BDO), are much higher compared to traditional non-biodegradable materials [14,15]. The introduction of various types of fillers of natural origin into PBS allows for a reduction in the final price of the obtained biocomposite and modification of its properties. Hence, researchers have great interest in biocomposites based on PBS with various natural fillers. The most frequently studied fillers are agricultural wastes, such as bran of wheat cereals [16,17,18], rice husk [19], wood flour or chips [20], ground coffee beans [21,22], wine grape pomaces [23], shells of various nuts [24,25,26], baobab fruit powder [27], cellulose, hemp, flax, sugar cane and bamboo fibers [28,29], hop fibers [30] and many others [4,31,32,33]. Other natural wastes such as oyster shell powder are used [34]. Research is also being conducted on the use of biochar as a filler for PBS [35,36].

In the available literature, there are no studies on the composting and aging nor on the antibacterial properties and cytotoxicity of PBS compositions with the addition of ground onion peels (OP) and durum wheat bran (*Triricum durum*) (WB). The previous work [37] presented composting studies of compositions with another variety of PBS and only common wheat bran; the composting took place in compost from a municipal composting plant, not in synthetic compost with a standardized composition [38]; and the composting time was limited to 70 days.

The presented work is a continuation of the research on PBS biocomposites with OP and WB. The results of the research on the physical, structural, thermal, thermomechanical and mechanical properties of these biocomposites are presented in [39].

In this research, we wanted to find how PBS biocomposites with OP and WB behave under the influence of selected environmental factors. We hypothesized that (1) natural fillers such as OP and WB accelerate the composting process of biocomposites based on PBS; (2) the content of OP and WB may have a negative effect on the resistance of biocomposites to aging, manifesting itself, among others, by a change in their color and surface morphology; (3) composites containing OP may exhibit antibacterial properties.

The aim of this work is to determine the effect of the type of filler of plant origin, OP and WB, and its content in a polymer biocomposite based on PBS on its biodegradability, resistance to artificial aging, susceptibility to fungal growth, antibacterial properties and cytotoxicity.

## 2. Materials and Methods

### 2.1. Materials and Production of Measurement Samples of Biocomposites

The tested biocomposites were made from the following ingredients:

− PBS for injection molding, named BioPBS with symbol FZ71 PB [40] (PTT MCC BIOCHEM Co., Ltd., Bangkok, Thailand) in the form of granules—density 1.26 g/cm^3^, melting point 115 °C, MFR (190 °C, 2.16 kg) 22 g/10 min.
− Onion peels (OPs) came from a local fruit and vegetable processing plant (Lublin, Poland) in the form of thin yellow-brown peels that were ground in a knife mill before being introduced into the biocomposite; particle diameter was up to 300 µm.
− Wheat bran WB from durum wheat (*Triricum durum*) came from PZZ Lubella GMW (Lublin, Poland) in the form of flakes; particle diameter was up to 500 µm.

A detailed description of the mentioned fillers, including, among others, particle morphology and filler density, was presented in the previous work [39].

The first stage of producing samples of the tested biocomposites was to obtain PBS granulates containing 15% and 30 wt.% OP and WB. The composition components were dried, then reconstituted and mechanically mixed in specific proportions. Biocomposite granulates were produced from the mixtures using a twin-screw extruder. From these granulates, dog-bone-shaped samples were finally produced using a screw injection molding machine in accordance with ISO 294-1:2017-07 [41]. Samples from pure PBS were produced directly from commercial granulate. Finally, five types of material samples were produced, designated as PBS (pure PBS without additives), OP15 (containing 15 wt.% onion peels), OP30 (containing 30 wt.% onion peels), WB15 (containing 15 wt.% bran) and WB30 (containing 30 wt.% bran). The machines used and the conditions of drying and processing of the biocomposite by extrusion and then injection molding are described in detail in [39].

### 2.2. Methodology

**Figure 1:**
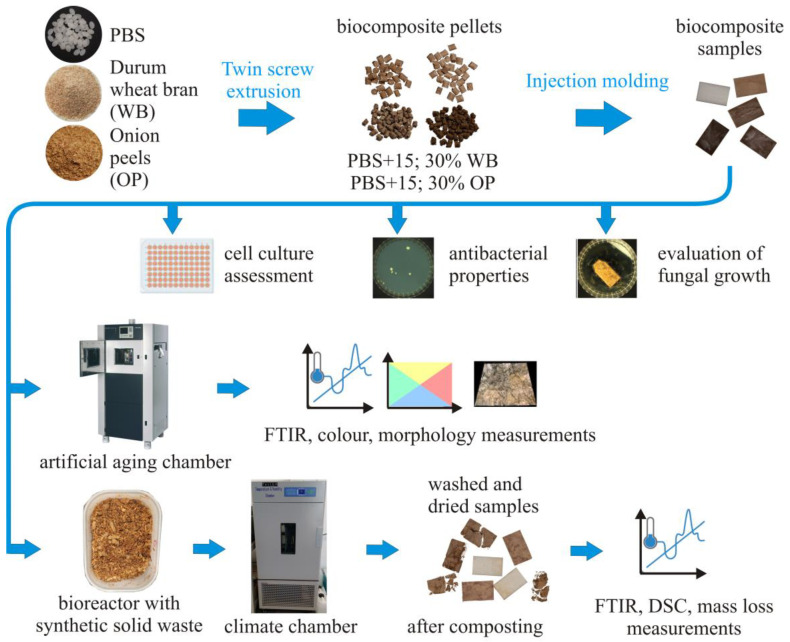
Diagram presenting the materials and methods used in this research.

The research program included tests to determine the effect of the content of 0–30 wt.% and the type of filler, onion peel (OP) and durum wheat bran (WB) in the PBS-based biocomposite on its biodegradability, resistance to artificial aging, fungal growth on the surface, cytotoxicity towards human skin cells and antibacterial properties. A diagram that graphically presents the materials and methods used in this research is shown in Figure 1.

The biodegradation of biocomposite samples was investigated on a laboratory scale under conditions simulating an intensive aerobic composting process. The method used determines the degree of disintegration of test materials according to ISO 20200:2015 [38]. The solid matrix used consists of synthetic solid waste inoculated with mature compost, which was obtained from a local waste management plant (Lublin, Poland). The synthetic waste used in this method was comprised sawdust, rabbit feed, ripe compost, cornstarch, saccharose, corn seed oil and urea in dry mass proportions consistent with the standard [38]. Biocomposite samples in the form of plates measuring 20 × 30 mm and 4 mm thick (obtained by cutting from a dog-bone shape) were placed in separate bioreactors (polypropylene containers) filled with synthetic solid waste. Bioreactors were kept in a chamber at a constant temperature of 58 °C and humidity of 60% (Climabox LHS-150HC-II from Agencja Anticorr, Gdańsk, Poland). According to the procedure described in the standard, at specific time intervals, the contents of the bioreactors were homogenized and water was supplemented to their initial mass, followed by 80% and 70% of the mass being supplemented. After the assumed biodegradation—composting time of 15, 30, 45, 60 and 90 days—the samples were extracted from the synthetic waste, washed and dried using a laboratory dryer at 60 °C, until a uniform mass was obtained. Samples after biodegradation were denoted by adding the suffix_15; _30, _45, _60 or _90 to their symbols, corresponding to the composting time.

Artificial aging of PBS and biocomposite samples was performed using the Xenotest Alpha+ artificial aging chamber (Atlas, Chicago, IL, USA). The conditions under which the samples were aged were in accordance with the recommendations of the standard ISO 4892-2:2013 [42]. For 1000 h, the samples were irradiated with a xenon lamp emitting radiation simulating solar radiation. An irradiance intensity of 60 W/m^2^ and a daylight filter system were used. The temperature in the chamber was maintained at 38 °C and 50% humidity. During irradiation, the samples were sprayed with distilled water for 18 min at time intervals of 102 min. The aging conditions used are close to the weather conditions prevailing in a temperate climate zone in the summer months.

Experimental studies of biocomposite samples subjected to the above-mentioned processes included the following:

- Mass loss of the biocomposites was determined based on the measurement of the dry mass of individual samples before and after the composting process. Mass loss was calculated from Equation (1), where *m_i_*—initial dry mass of the sample; *m_r_*—dry mass of the remaining sample after composting that has not decomposed.

(1) $$\mathrm{mass}\;\mathrm{loss}=\left(\frac{m_{i}-m_{r}}{m_{i}}\right)\times 100\%$$

- FTIR analysis of the samples was performed using a TENSOR 27 FTIR spectrophotometer (Bruker, Billerica, MA, USA) equipped with an ATR (Attenuated Total Reflectance) attachment with a diamond crystal. The spectra (32 scans per spectrum) were collected for a range of 600–4000 cm^−1^ and a resolution of 4 cm^−1^.
- Differential scanning calorimetry (DSC) testing of the samples was made using a 204 F1 Phoenix DSC scanning calorimeter (NETZSCH, Günzbung, Germany) and NETZSCH Proteus data processing software version 6 (NETZSCH, Günzbung, Germany), in accordance with the standard ISO 11357-1: 2023 [43]. DSC thermograms were recorded for the following cycles: heating (I) from −100 °C to 140 °C (at a rate of 10 °C/min), cooling from 140 °C to −100 °C (at a rate of 10 °C/min) and heating (II) from −100 °C to 140 °C (at a rate of 10 °C/min). The samples, weighing approximately 10 mg, were tested in aluminum crucibles with a pierced lid. DSC curves were used to determine the degree of crystallinity *X_c_*, melting enthalpy Δ*H_m_*, melting point *T_m_*, crystallization temperature *T_c_* and glass transition temperature *T_g_* for the obtained composites. The degree of crystallinity was calculated from the relationship presented in Equation (2):

(2) $$X_{c}=\left(\frac{∆H}{\left(1-u\right)\times {∆H}_{100\%}}\right)\times 100\%$$

- assuming that for PBS, Δ*H*_100%_ = 110.3 J/g; *u*—share of the organic filler. The *u* parameter for composted samples was calculated based on thermogravimetric curves. The DSC curve inflection point in the glass transition area was adopted as the glass transition temperature.
- The color of the samples was determined using an X-Rite Ci4200 spectrophotometer in accordance with the ASTM E308 standard [44]. The CIELab system was used to describe the color, in which it is defined in the L*, a*, b* space. The *a* parameter describes the color from green (negative values) to red (positive values); *b* parameter—the color from blue (negative values) to yellow (positive values). The *L* parameter is luminance—lightness, representing the gray scale from black to white (value 0 corresponds to black, and 100 to white). The difference between two colors, which in this system correspond to two points in the three-dimensional L*, a*, b* space, is described by the following relationship:
  (3) $$∆E=\sqrt{{∆L}^{2}+{∆a}^{2}+{∆b}^{2}}$$
  Δ*L*, Δ*a* and Δ*b* denote the difference in color parameters between the compared samples, respectively.
- The surface and fracture photos of the samples were taken using a NIKON Eclipse LV100ND optical microscope equipped (Tokyo, Japan) with a DS-U3 camera with NIS-Elements AR 4.20.00 software. The reflected light method was used for observation. The black field method was used for fracture photos, and the focus stacking method was used to obtain a large depth of field. The bright field observation method and 3D photo stacking were used to take surface photos of the samples. The compared samples were observed at the same magnification and illumination values.
- The tests of fungal growth on the studied biocomposites were performed based on the ISO 846:2019 standard (Method A) [45]. According to the standard, the inoculum used was a mixture of five reference strains of fungi: *Aspergillus niger* (ATCC 6275), *Penicillium pinophilum* (ATCC 36839) syn. *Talaromyces pinophilus*, *Paecilomyces variotii* (ATCC 18502), syn. *Byssochlamys spectabilis*, *Trichoderma virens* (ATCC 9645) and *Chaetomium globosum* (ATCC 6205). According to the standard, the samples were divided into three test batches: batch 0—control samples, stored in standardized climatic conditions of conditioning and testing according to ISO 291:2008 [46] (23 ± 1 °C, 50 ± 5% RH); two fragments/sample were used; batch S—sterile samples, stored in the same conditions as test batch I; two fragments/sample were used; batch I—test samples inoculated with microorganisms and incubated; five fragments/sample were used. The samples were incubated for 28 days at 29 ± 1 °C. Visual evaluation was performed using a SCAN 1200 automatic colony counter (Interscience, Saint-Nom-la-Breteche, France).
- Cell culture experiments were conducted using a normal human skin fibroblast BJ cell line (ATCC, London, UK). The cells were cultured in EMEM supplemented with 10% FBS (Pan-Biotech GmbH, Aidenbach, Bavaria, Germany), 100 U/mL of penicillin and 100 µg/mL of streptomycin (Sigma-Aldrich Chemicals, Warsaw, Poland) at 37 °C in a humidified atmosphere of 5% CO_2_ and 95% air (Heraeus Cytoperm 2, Thermo Scientific, Waltham, MA, USA). The cytotoxicity test was performed by an indirect method using material extracts according to the ISO 10993-12:2012 standard [47]. The sterilized materials (ethylene oxide sterilization) were incubated in EMEM culture medium with 2% FBS for 24 h at 37 °C, in a ratio of 1.25 cm^2^/mL of culture fluid. Simultaneously, cells at a density of 2 × 10⁵ cells/mL were seeded in a 96-well plate in a volume of 100 µL and also cultured for 24 h at 37 °C. After this period, the medium was removed from the cells and replaced with the material extracts. The negative cytotoxicity control (sample designated as “control”) consisted of cells cultured exclusively in culture medium (EMEM with 2% FBS). The prepared cells were then incubated for an additional 24 h under the same conditions. After this incubation, an MTT colorimetric assay was performed (Sigma-Aldrich Chemical, Warsaw, Poland). MTT solution (1 mg/mL) was prepared in culture medium, and 100 µL of this solution was added to each well containing cells, followed by incubation for 3 h at 37 °C. During this period, mitochondrial dehydrogenase enzymes in viable cells reduced MTT to formazan, resulting in the formation of formazan crystals within living cells. After incubation, 100 µL of a 10% SDS solution in 0.01 M HCl was added to each well to dissolve the formazan crystals. After 12 h, the absorbance of the formazan solution was measured at a wavelength of 570 nm using a plate reader. The amount of formazan formed was proportional to the number of viable cells. MTT test results were expressed as a percentage of the optical density (OD) value obtained relative to the negative cytotoxicity control.
- Antibacterial activity evaluation was performed based on the standard AATCC Test Method 100-2004, as previously described [48]. Reference bacterial strain *Escherichia coli* (ATCC 25922) was grown at 37 °C for 24 h in Mueller–Hinton Agar medium (Biomaxima, Lublin, Poland). Then, the bacteria were scraped and suspended in sterile Mueller–Hinton broth to a density of 0.5 McFarland. Briefly, the materials (previously sterilized with ethylene oxide) were placed in sterile Petri dishes and treated with 50 µL bacterial suspension diluted to a concentration of 1.5 × 10⁴ CFU (prepared in 250-fold-diluted Mueller–Hinton broth). All samples were incubated at 37 °C for 24 h and then transferred into an appropriate volume of sterile 0.9% NaCl solution. In the next step, the samples were vigorously shaken for 60 s to remove bacteria from the materials. Simultaneously, a bacterial growth control was prepared by incubating the same volume of the bacterial suspension without contact with tested materials. All samples were then plated on Mueller–Hinton agar using an automated plater (EasySpiral Dilute, Interscience, Saint-Nom-la-Bretéche, France). The plates were incubated at 37 °C for 24 h. Next, CFUs were counted for each plate using the Scan 300 counter. Additionally, representative images of bacterial growth on the plates were taken.

The results obtained are presented in the form of graphs, where the mean values are marked together with the standard deviations.

## 3. Results and Discussion

### 3.1. Composting

#### 3.1.1. Mass Loss

**Figure 2:**
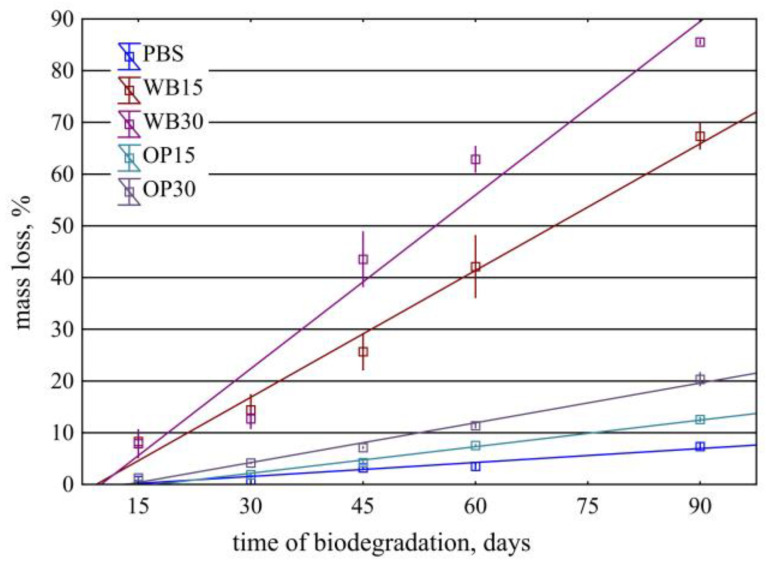
Mass loss of PBS and its composites depending on the time of biodegradation in compost.

The influence of the mass content of onion peel and bran on the mass loss of biocomposite moldings as a result of biodegradation–composting is shown in Figure 2. The dependence of the mass loss of samples on the biodegradation time can be described using linear functions. This is indicated by high values of the determination coefficients of the determined linear regression equations and the fulfillment of the required assumptions by all of them (significance of the equation and regression coefficients, normality of the distribution of residuals, randomness of their deviations and non-biasability, and lack of autocorrelation).

**Figure 3:**
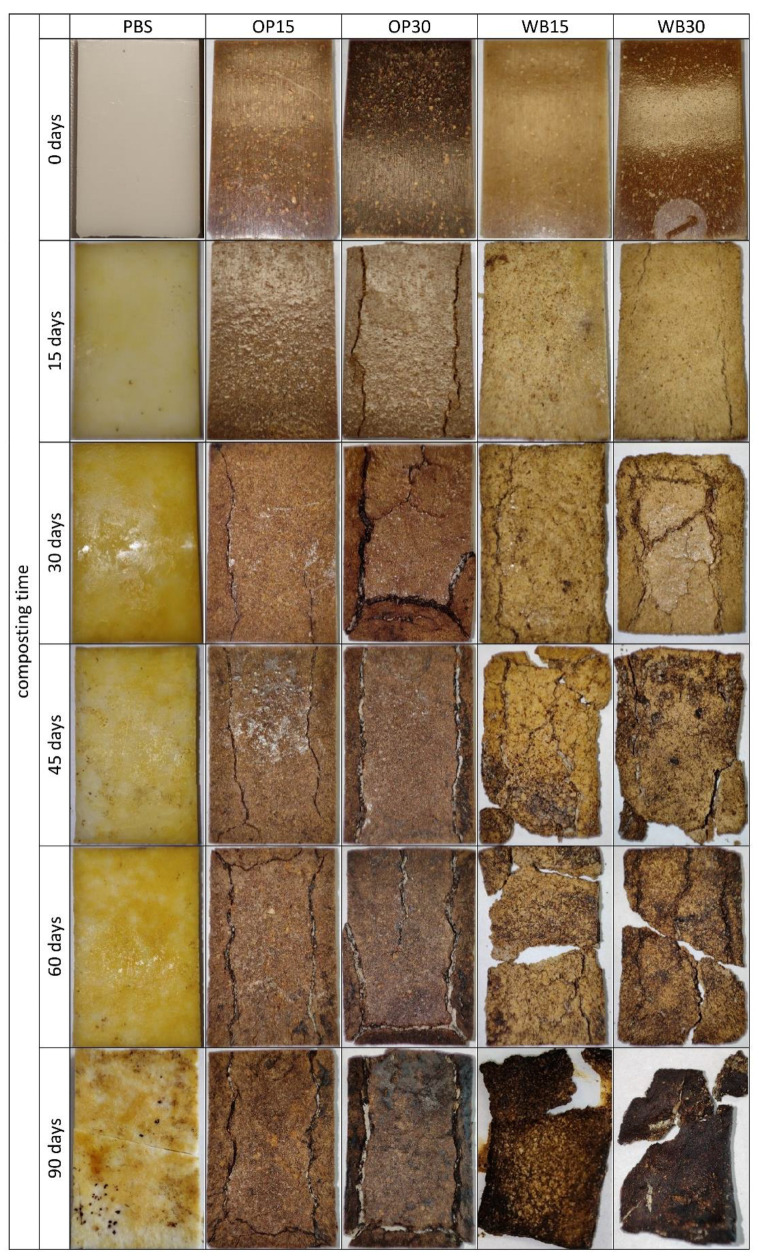
Photos of biocomposite samples depending on the composting time and the content of OP or WB.

On the surface of the PBS samples at the initial stage of biodegradation, pits appear, which are the effect of microorganisms’ activity (Figure 3). As the composting process progresses, the shallow pits deepen, creating spherical depressions. From the 45th day of composting, a network of microcracks is visible on the surface, which may be the effect of the increase in the degree of crystallinity and the development of internal stresses in the sample. After 90 days of composting, deep pits and a dense network of microcracks appear on the surface of the samples, some of which lead to sample fragmentation. This is a typical course of PBS biodegradation also described in the literature [49]. However, the mass of samples from PBS alone changed very little during composting. After 90 days of composting, the average mass loss was 7.35%, which indicates a small loss of 0.08% for each day of composting. A similar slow course of PBS biodegradation was also described by Puchalski at all [50]. The performed regression analysis allowed for describing this relationship with a linear function, m = 0.090 × time − 1.177 ± 0.792, for which the coefficient of determination was R^2^ = 0.90.

**Figure 4:**
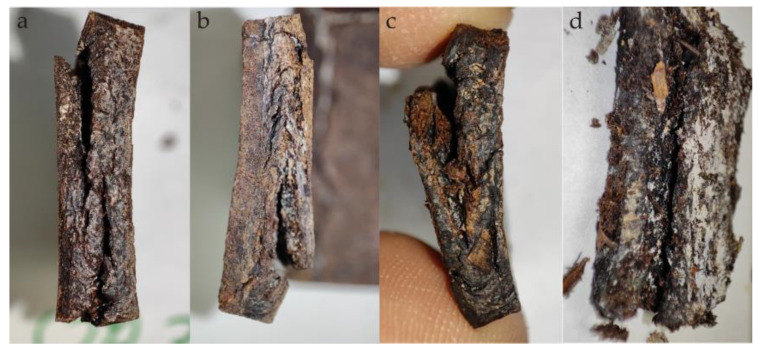
Side view of samples after composting: (**a**) OP30_45, (**b**) OP30_60, (**c**) OP30_90 and (**d**) OP30_90 directly after extracted from the synthetic waste.

**Figure 5:**
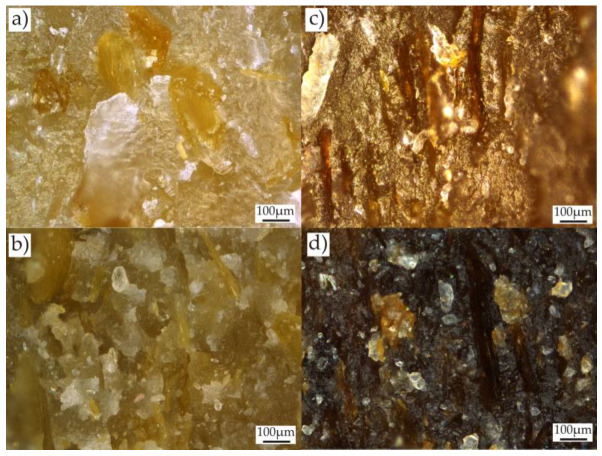
Microscopic images of sample cracks perpendicular to the flow direction of the biocomponent in the injection mold: (**a**) WB15, (**b**) WB30, (**c**) OP15, (**d**) OP30.

In the case of samples containing OP, filler particles are visible directly under the surface or on their surface. In the initial stage of composting, as a result of water absorption, they are clearly visible on the surface of the samples (Figure 3). After 30 days of composting, these samples have a clearly damaged surface and exposed filler particles. Additionally, there are deep longitudinal cracks in such samples, which enlarge during composting and then lead to sample delamination (Figure 4). As a result, microorganisms can more easily penetrate the interior of the samples and the surface they affect increases. The observed cracks, similar to those in pure PBS, may be the result of an increase in the degree of crystallinity and the creation of internal stresses in the sample. Sample delamination, on the other hand, can be associated with the longitudinal orientation (in the direction of the flow of the material in the mold) of onion peels, which are plate-shaped, visible on the transverse fractures of the samples (Figure 5). The parallel arrangement of the filler plates affects the delamination of the samples in a plane parallel to their surface.

The increasing amount of OP in the biocomposite increased the rate of its biodegradation. The mass of the biocomposite samples containing 15% of OP after 90 days of composting decreased by an average of 12.57%, which corresponds to an average loss of 0.14% for each day of composting. This relationship can be presented as a function, m = 0.174 × time − 2.978 ± 0.687, for which the coefficient of determination R^2^ = 0.98. In the case of biocomposite samples containing 30% of OP, after 90 days, the average mass loss was 21.34%; therefore, the degradation proceeded at a rate of 0.24% for each day of composting. In this case, the relationship is well described by the function m = 0.252 × time − 3.311 ± 0.957, where R^2^ = 0.98.

The largest mass losses during composting were observed in the case of biocomposites containing bran. Bran particles are visible on the surface or directly below the surface of the input samples for composting. After only 15 days of composting, these samples have a clearly disturbed surface—a highly porous structure (suggesting polymer disintegration) and exposed bran particles that were originally located in deeper parts of the samples. Bran, due to its chemical structure, is preferred by microorganisms [51] and is more easily enzymatically hydrolyzed compared to OP. It was degraded first, exposing deep areas of the polymer matrix, allowing for microorganisms to colonize them. Similarly to the use of OP, cracks in such samples grew during composting, facilitating the penetration of water and microorganisms (Figure 2). However, there was no delamination of the samples, due to the lack of visible bran orientation in the cross-section of the samples (Figure 5). After 90 days, the mass of such compositions was lower on average by 67.34% at 15% of WB content and by as much as 85.54% at 30% of WB content. The nature of these changes is described by linear functions m = 0.8176 × time − 7.671 ± 6.064 and m = 1.1196 × time − 11.24 ± 7.887, for which R^2^ = 0.93 and R^2^ = 0.93, respectively. A similar increase in the biodegradation rate of PBS compositions with other natural fillers was observed in previous works [19,52,53].

#### 3.1.2. DSC

**Figure 6:**
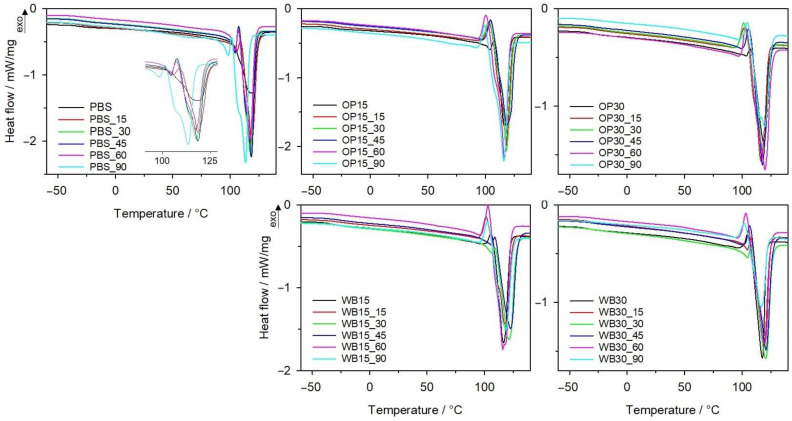
DSC thermograms of II heating of PBS and its biocomposites with WB and OP after specified intervals of composting.

In order to find the structural changes in biocomposites due to composting, samples after each interval degradation in synthetic waste were tested using the DSC method. In Figure 6, DSC thermograms of PBS, OP and WB composites from the second heating scan are presented. From the first heating scan, water evaporation from samples was observed between 80 and 110 °C. As can be seen from the data in Table 1, the composting process clearly influenced the *T_g_* and *T_m_* of the PBS. With increased composting duration, the *T_g_* of PBS increased from −32 °C to −24 °C after 90 days of composting. This increase is related to the increase in the degree of crystallinity observed with the course of biodegradation. Two melting endothermic peaks on the DSC thermogram of degraded PBS are observed, and additionally, the larger ones are not symmetrical; with progressive biodegradation, they become wider, with a visible shoulder. A significant drop in *T_m_* from 104/117 °C to 98/113 °C took place after composting. This suggests that crystallites with different sizes are formed during biodegradation. Moreover, changes in crystallinity degree were observed. Increasing the time of composting caused an increase in *X_c_*, which reached 95% after 90 days. The same trend of *X_c_* changes was also observed for composites with OP and WB, regardless of the mass of natural filler. This observation is convergent with previously published results for PBS, other polyesters and their composites [37,54,55], and confirms that during composting, the amorphous phase of polymer is firstly degraded. As can be seen from Figure 6, the course of DSC curves for composites are different from that of pure PBS. The melting peaks of the PBS component became wider with increasing biodegradation time; for OP30, after 90 days, an even, bimodal peak was observed. This is due to the presence of crystallites of different sizes. Most likely, biodegradation caused the polymer chains’ scission and thus facilitated the formation of the crystalline phase.

#### 3.1.3. Changes in Chemical Structure

**Figure 7:**
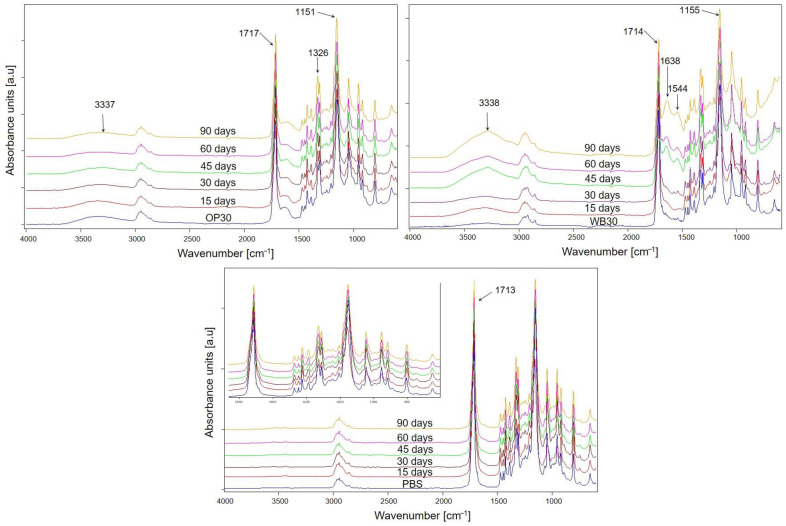
FTIR spectra of studied materials after specified intervals of composting.

FTIR analysis was applied to determine changes in the chemical structure of composted materials. The spectra of composite samples (shown in Figure 7) reveal greater changes than those of pure PBS compared to the initial ones; in particular, significant differences are visible for WB30 biocomposite. The ester bonds present in the backbone of PBS are prone to hydrolysis under composting conditions [56], which leads to the cleavage of the polymer chains and a reduction in polymer molecular mass [16]. As a result of hydrolysis, hydroxyl groups are formed at the ends of polymer chains; hence, an increase in the intensity of absorption bands originating from the stretching vibrations of -OH groups at about 3338 cm^−1^ is observed in the spectra [57]. This increase is particularly visible in the spectra of the WB30 composite, which contains a large share of bran in its structure. This sample showed the highest mass loss after composting, which was caused by the susceptibility of the bran to microorganisms and consequently contributed to the faster decomposition of the polymer matrix. Moreover, in the spectra of WB30, an increase in the intensity of the absorption band at about 1638 cm^−1^ is observed with the progress of composting, which can be attributed to the bending vibrations of HOH in water [58,59], which could have been absorbed by cellulose or organic biodegradation products. A new band at 1544 cm^−1^ also appeared, which may be due to the vibrations of the amide group [60] formed during the enzymatic hydrolysis of bran [51,52]. On the other hand, only slight changes were observed in the spectra of pure PBS, mainly in the carbonyl band at 1713 cm^−1^ [57], which became broader as composting proceeded. Moreover, for all tested materials, one more difference in the intensity of the carbonyl bands at about 1714 cm^−1^ and the C-O-C asymmetric stretching vibrations band at about 1151 cm^−1^ is visible [57]. As composting progressed, a slight increase in the intensity of the C=O band was observed relative to the intensity of the C-O-C band. This may confirm the breakdown of the ester bonds by hydrolysis and the formation of a carboxyl group.

### 3.2. Evaluation of Fungal Growth

**Figure 8:**
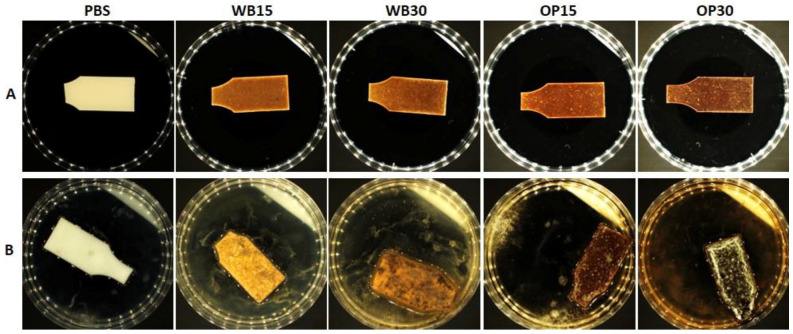
Illustrative photos of the visual evaluation of batch I samples: (**A**) before incubation; (**B**) after incubation.

According to the applied methodology based on ISO 846:2019 Method A, the test samples were exposed to a suspension of a mixture of fungal spores in the presence of humidity ≥ 95% relative humidity. After exhaustion of the limited nutrients stored in their own cells, the fungi can only grow at the expense of the material contained in the samples. Fungi grew the least in the PBS sample. For the composite samples, there was a visible relationship between the content of natural components (onion peel and bran) and fungi growth. The more natural the component, the more intensive the fungi growth. In each variant, fungi growth covered a larger sample surface than in the PBS sample (Figure 8). On samples containing bran, fungi covered up to 50% and over 75% of the surface of samples WB15 and WB30, respectively (Table 2). On samples containing onion peel, fungi covered up to 50% and 100% of the surface of samples OP15 and OP30, respectively.

The intensive growth of fungi on the surface of samples containing natural components, in accordance with the assumption of the standard, indicates the use of the polymeric material as a source of nutrients. Fungi are known to have the ability to enzymatically hydrolyze starch, cellulose and lignocellulosic materials, which provide them with a source of readily available carbon [61]. For this reason, fungal growth observed on composite samples with plant-derived fillers was significantly greater than in pure PBS. Furthermore, the hydrolysis of OP or WB advances the fungi enzymes, especially esterases [62,63], to react with PBS. Previous studies also reported a significant acceleration in the fungal hydrolysis of polymers when blended with starch or cellulose, e.g., polyesters PBS–cellulose, PLA–starch or PCL–starch or even polyolefins starch–PP, starch–PE or PVC–cellulose [64]. Based on the obtained results, it can be assumed that WB and OP were an accessible source of energy for fungi, increasing the potential for biodegradation in comparison to the neat PBS material.

### 3.3. Artificial Aging

#### 3.3.1. Color

**Figure 9:**
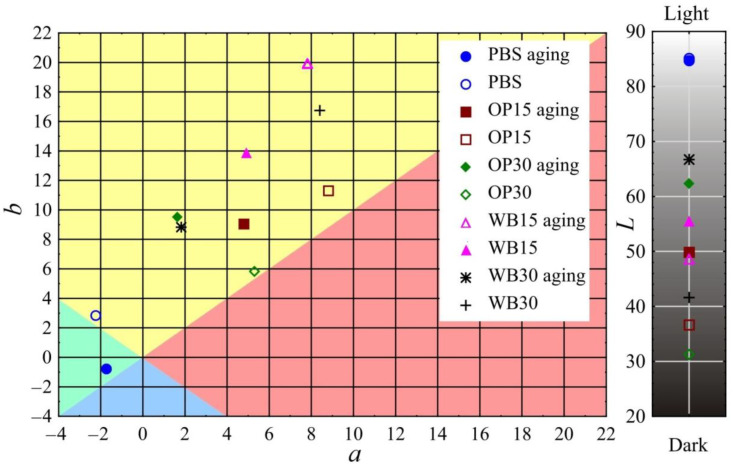
Color parameters *L*, *a* and *b* before and after artificial aging of biocomposite samples.

**Figure 10:**
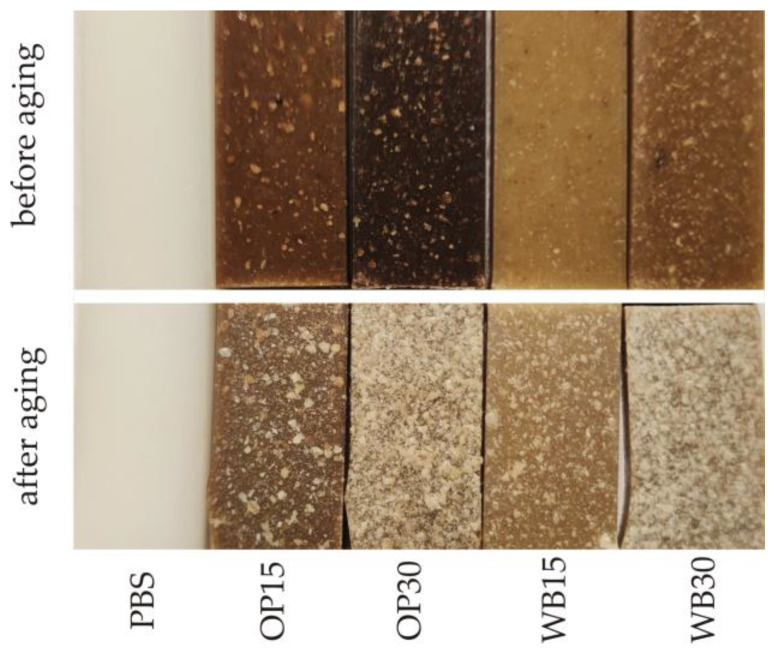
Images of the tested biocomposites samples before and after artificial aging.

The results of the color measurement of the biocomposite samples before and after the artificial aging process are presented in Figure 9. In the case of all the tested samples, the color change due to aging is at least distinct and usually high. The smallest color change was observed in the pure PBS samples. The determined color difference value ∆*E* was 3.7 and was similar to that obtained for another PBS variety in previous studies [16]. It should be considered clearly noticeable, even by an inexperienced observer, because in this type of study, the range (3.5 < ∆*E* < 5) is assumed to be such. The lightness *L* of the PBS samples did not change, while the value of the *b* parameter dropped significantly, which corresponds to a color change towards *a* blue shade. The greatest color changes due to aging among all the tested samples occurred in the biocomposites containing OP. In the OP15 samples, the change was ∆*E* = 14.0, while in the case of the OP30 samples, it was as much as ∆*E* = 31.5, which classifies it as high (∆*E* > 5). As a result of the aging process, the samples containing OP mainly brighten/fade, as evidenced by the significant increase in their luminance *L* (for OP15, an increase of 13.2; for OP30, an increase of 31.1). This is the effect of visible brightening/fading of the OP particles located on the surface or directly under the surface of the samples, which is visible in Figure 10. At the same time, in the case of the OP30 samples, the parameter *a* decreased by the same absolute value |3.7| (change towards shades of green) and the parameter *b* increased (change towards shades of yellow). In the case of the OP15 samples, the value of both parameter *a* (change towards shades of green) and parameter *b* (change towards shades of blue) decreased. The decrease in the *b* parameter in these samples should be associated with a significantly higher share of the PBS matrix in relation to the OP particles on their surface (Figure 10). The color of pure PBS, as described above, changed towards shades of blue. Slightly smaller color changes, but of a similar nature, were observed in the case of samples containing WB. As a result of artificial aging, the color change in WB15 samples was ∆*E* = 9.6, while in WB30 samples, ∆*E* = 27.1 was obtained, which classifies the changes as high (∆*E* > 5). In these samples, there was also a brightening/fading of WB particles located both on the surface and directly under their surface (Figure 10). As a result, the luminance *L* increased by 6.9 and 25.1 for WB15 and WB30, respectively. However, greater changes in the remaining color parameters were observed in comparison to the samples with OP. The parameter a decreased by 2.9 and 6.6 for WB15 and WB30, respectively, i.e., there was a change towards shades of green. On the other hand, the decrease in parameter *b*, indicating a change in color towards shades of blue, was even greater and amounted to 6.1 and 7.9 for WB15 and WB30, respectively. It should also be noted that the color difference in samples OP30 and WB30 amounting to ∆*E* = 4.5 results mainly from the different luminance *L*, because the remaining parameters *a* and *b* are very similar.

The described color changes in samples containing both OP and WB are related to the degradation of their main building components, lignin and cellulose. Onion peels mainly consist of α-cellulose and lignin, as well as hemicellulose and extractives, phenolic compounds, flavonoids and flavones [65]. The main components of wheat bran WB are also cellulose, lignin, hemicellulose, as well as phytic acid, oligosaccharides, non-starch polysaccharides, fats and proteins [66,67]. Lignin exhibits hydrophobic properties and is therefore quite resistant to degradation in water [68,69]. In the artificial aging chamber, the samples were subjected to water spray combined with simultaneous UV irradiation, additionally at elevated temperature. In such conditions, lignin is susceptible to photodegradation. As a result of the depolymerization of lignin, hydrophilic cellulose is exposed. Both OP and WB particles swell, which causes deeper radiation penetration and, as a result of photodegradation, the oxidation reaction in artificial conditions [70,71]. Moreover, onion peels’ color is derived from the phenolic compounds, flavones and flavonoids that possess antioxidant properties [72]. Since these compounds show significant absorption of UV light, OP composites showed the greatest change in color during the artificial aging tests [73].

#### 3.3.2. Morphology

**Figure 11:**
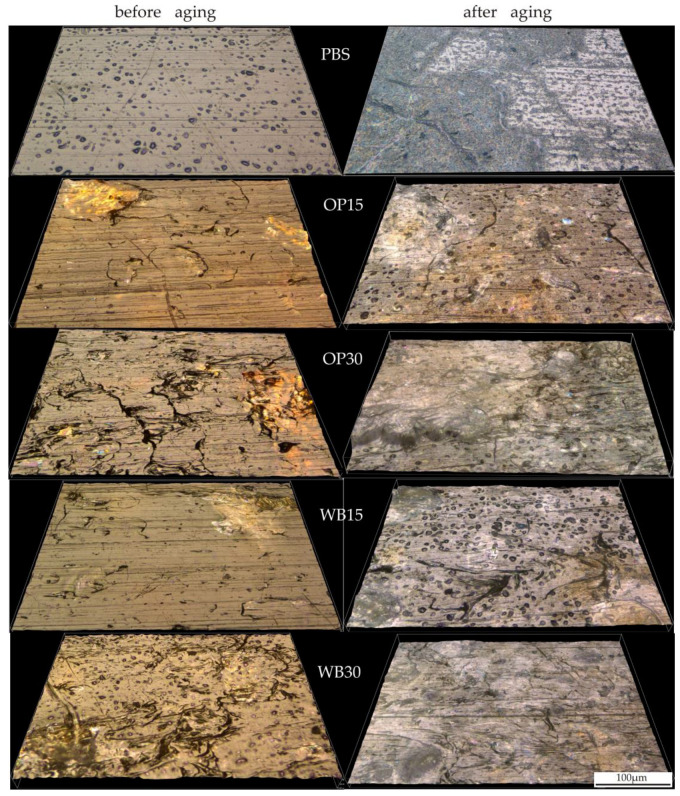
Surface appearance of the tested biocomposites samples before and after artificial aging.

Morphological changes in samples subjected to artificial aging were observed using an optical microscope in reflected light using the 3D photo stacking method. The obtained three-dimensional images of the surface topography of the samples before and after aging are shown in Figure 11. The surface of the pure PBS samples underwent the smallest changes as a result of artificial aging. However, it is possible to distinguish areas where its roughening is clearly visible, and to see the scratches that have become larger.

The surface of the biocomposite samples before aging shows a clear increase in roughness with increasing OP and WB content. Particles of both fillers are visible on the surface of the samples, and those located directly on the surface are distinguished on it as bulges. Small cracks and grooves resulting from the presence of filler particles are also visible. The increase in roughness with the content of fillers is typical for PBS composites with powder fillers [74,75] and is the result of the increase in the number of filler particles located directly on the surface of the sample. As a result of artificial aging, the surface of the biocomposites was significantly degraded. The surface roughness of the samples increased significantly, and the trend of these changes results from the effect of the filler content on roughness observed before aging. The surface of the samples became more porous, and the cracks enlarged. The changes that occurred on the surfaces of the samples containing OP and WB are similar and should be related to the analysis of the results concerning the color. The heterogeneity of the sample surface visible in the photos is mainly the result of the degradation of the fillers and the mechanical washing out of the degradation products by the water sprayed during aging. This causes the surface of the PBS matrix to be exposed. The photos of the OP15 and WB15 samples as well as partly OP30 show exposed areas with a surface similar to that observed for pure PBS before aging.

#### 3.3.3. Changes in Chemical Structure

**Figure 12:**
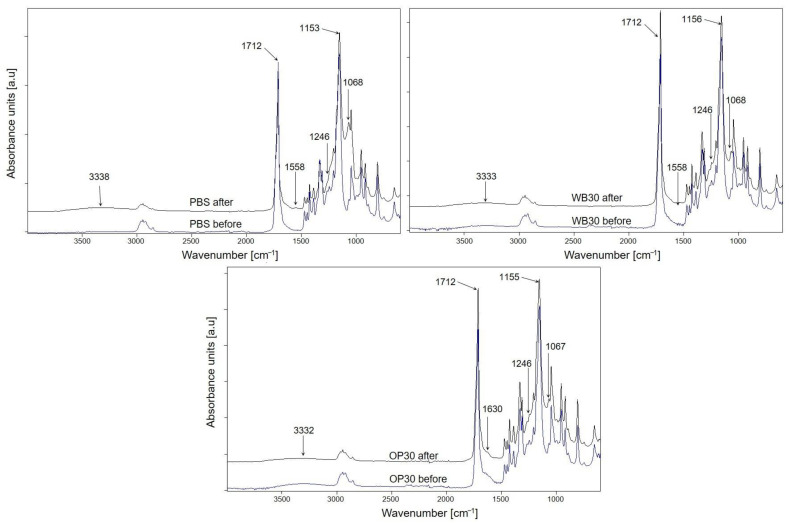
ATR-FTIR spectra of PBS, WB30 and OP30 before and after 1000 h of artificial aging.

During artificial aging, tested samples were exposed on UV irradiation and periodically to water, which could lead to photodegradation of both PBS and fillers such as WB and OP. Taking into account the FTIR spectra of the PBS, OP30 and WB30 biocomposites before and after aging (Figure 12), some changes are visible. For each sample, a new broad band at about 1060 cm^−1^, corresponding to the C-O(H) stretching vibration in the alcohol group, and band widening at 1246 cm^−1^ (derived from O-H deformation vibration) are present after aging [57]. Additionally, a significant increase in the intensity of the carbonyl group vibration band at 1712 cm^−1^ in relation to the band at 1155 cm^−1^ (C-O-C asymmetric stretching vibrations) after aging of composites took place. This change in the spectra of PBS is of the opposite character. The reduction in carbonyl band intensity may be the result of a decarboxylation reaction following the cleavage of the ester bond [76]. Moreover, in the spectrum of PBS after aging, a new band at 3338 cm^−1^ appeared, which was the result of stretching vibrations of the hydroxyl group [57], while the intensities of these bands in the spectra of aged biocomposites increased in comparison to the initial spectra. These changes indicate that photooxidation occurred in the structure of the tested samples, leading to the cleavage of polymer chains [16,76,77]. The decrease in the intensity of the C-O-C band and simultaneous increase in the intensity of the carbonyl band testify that the ester bonds were broken. It is also possible that carboxyl groups were formed, as a band at 1558 cm^−1^ (carboxylate group stretching vibrations) [7] is present in the spectra of PBS and WB30. On the basis of the obtained FTIR results, it can be concluded that photooxidation of tested samples run through α-hydrogen abstraction and the Norrish I chain cleavage reaction, as was postulated in earlier studies [76,78,79]. The β-hydrogen abstraction leading to the formation of unsaturated compounds is unlikely in case of PBS, OP and WB composites since no absorption bands of unsaturated C=C groups are observed in the FTIR spectra.

### 3.4. Cell Culture Assessment

**Figure 13:**
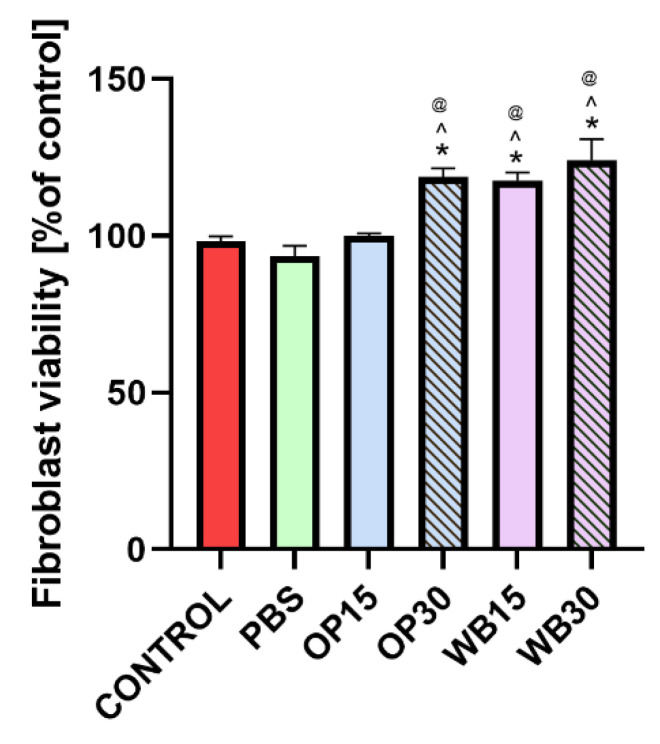
Fibroblast viability after 24 h incubation with extracts obtained from PBS, OP15, OP30, WB15 and WB30 materials. The results were obtained using the MTT assay. (*) symbol indicates statistically significant results between control and the samples; (^) symbol indicates statistically significant results between PBS and the samples; (@) symbol indicates statistically significant results between WB15 and the samples, according to one-way ANOVA with post-hoc Tukey’s test (*p* < 0.05).

The conducted in vitro cytotoxicity studies aimed to confirm the biological safety of PBS biocomposites with the addition of natural fillers, such as onion peels and durum wheat bran. The results of the MTT assay showed that none of the tested materials exhibited cytotoxicity toward the BJ skin cell line, as cell viability exceeded 70% compared to the control. This indicated no negative impact of tested samples on skin cells (Figure 13). Importantly, neat PBS polymer is also non-toxic, as was confirmed in studies using murine fibroblast (L929 cell line), which further emphasizes its biocompatibility as a base material [80]. Furthermore, the addition of plant-derived fillers, particularly in materials labeled WB15 and WB30, enhanced the viability of human skin cells (exceeding 100% compared to the control). These findings are not surprising because both onion skin and wheat bran are biologically safe components of human food [81,82]. The results of this test confirm that the tested biocomposites are biologically safe and may have broad applicability.

### 3.5. Antibacterial Properties

**Figure 14:**
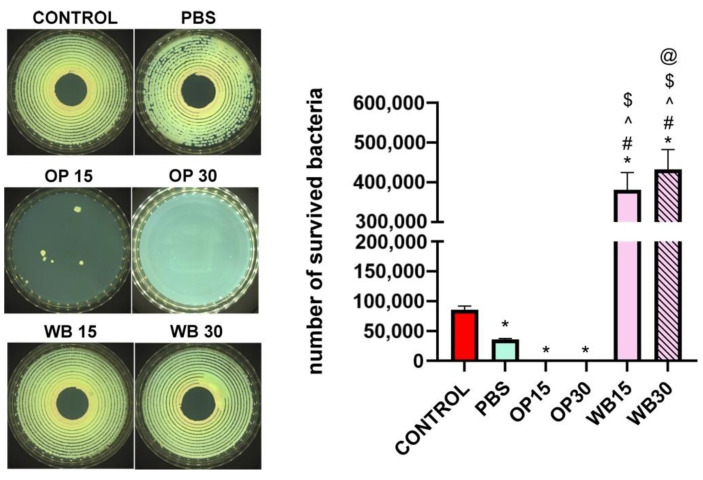
Viability of *E. coli* in antimicrobial test according to AATCC 100-2004 standard, where (*) symbol indicates statistically significant differences between the samples and the control for this test; (#) symbol indicates statistically significant differences between PBS and the samples; (^) symbol indicates statistically significant differences between OP15 and the samples; ($) symbol indicates statistically significant differences between OP 30 and the samples; (@) symbol indicates statistically significant differences between WB15 and the samples, according to one-way ANOVA post-hoc Tukey’s test (*p* < 0.05). The representative images illustrate bacterial growth on Mueller–Hinton agar following the experiment, in which the materials were incubated with the bacterial suspension.

The test showed that materials with durum wheat bran (WB 15 and WB 30) did not inhibit but enhanced the growth of *E. coli* bacteria in comparison with the control inoculate (Figure 14). In turn, the OP 15 and OP 30 materials containing onion peels in varying content effectively inhibited the growth of bacteria. However, OP 30 did not allow for the bacteria to grow ultimately, while OP 15 enabled the growth of single bacterial colonies, showing that the concentration of OP incorporated into the composite sample is significant for achievement of full antibacterial efficacy. Moreover, it is known from other studies that extracts obtained from onion peels exhibit both antibacterial and antifungal activities [83]. Overall, it seems obvious that onion peels incorporated into polymer matrix provide high and efficient antibacterial protection in direct contact with bacteria and indicate their high antibacterial potential in design of multifunctional polymers.

## 4. Conclusions

The analysis of the obtained research results showed a significant influence of the content of natural fillers OP and WB on the analyzed properties, as well as the occurrence of differences in the intensity of this impact.

It was shown that the mass loss of samples of both pure PBS and its biocomposite with OP and WB linearly depends on the composting time. Composting of pure PBS proceeds slowly; as a result, after 90 days, its mass loss was only 7.35%. Introducing natural fillers to the composition significantly accelerates the biodegradation process, which depends on their content. The biocomposite containing 15% OP after 90 days of composting showed 12.57% mass loss, and that containing 30% OP already 21.34%. The biodegradation of samples containing WB proceeded the fastest. In the compared period, their mass loss was 67.34% with the content of 15% WB and as much as 85.54% with the content of 30% WB. The observed acceleration of composting is related to the fact that natural fillers are the first to biodegrade and expose deeper areas of the polymer matrix to the action of microorganisms and water. This was also confirmed by FTIR analysis; the most noticeable changes in the chemical structure after composting were detected in the case of WB composites. Under composting conditions, PBS was partially hydrolyzed, which led to the cleavage of the polymer chains. As a consequence, an increase in the degree of crystallinity of pure PBS and composites was observed.

Artificial aging clearly affects the color change in pure PBS, and in the case of the tested biocomposites, the color change is high. The color change in samples containing OP and WB is the result of photodegradation of lignin, which is a component of both fillers and leads to their brightening/fading, which is manifested by a significant increase in luminance L due to the decomposition of the chromophore. The color change of OP samples is greater than that of WB. Changes in the FTIR spectra after aging confirm that photooxidation reactions took place when samples were irradiated with UV light.

The degradation of fillers due to artificial aging was also the main cause of changes in the surface morphology of biocomposite samples.

The assessment of fungal growth on the surface of the samples confirms the potential of the biocomposite for biodegradation and the possibility of using the material as an energy source for the intensive development of microorganisms.

Antibacterial studies demonstrated that materials supplemented with OP exhibited high antibacterial properties against the reference strain of *E. coli*, compared to materials enriched with WB, which allowed for bacterial growth at a level even higher than control. In contrast, cytotoxicity studies on human skin cells revealed that all tested materials were non-toxic. This indicates that tested composites, in particular those containing OP, are highly promising for productions of non-toxic and biodegradable polymers of high antibacterial activity.

The high content of the tested OP and WB fillers in biocomposites, apart from significantly reducing the material costs of their production, may have a beneficial effect on the time of disposal through composting of utility items made from them. The high degree of decomposition indicates that the proposed biocomposites are compostable. Considering the shorter biodegradation time, composites containing WB exhibit better properties. However, if the application requires antibacterial properties, only composites with OP can provide them. Both fillers limit the use of composites in applications where they are exposed to weather conditions and it is important to maintain an unchanged color. To help determine the potential applications of composites, further research is planned, including the examining effect of aging on their strength properties and determining the resistance of products made from them to mechanical dishwashing.

## Figures and Tables

**Table 1:** Changes in melting point (*T_m_*), crystallization (*T_c_*) and glass transition (*T_g_*) temperatures; the enthalpy of melting (Δ*H_m_*); and degree of crystallinity (*X*_c_) of tested materials after specified intervals of composting.

Sample	Heating I			Cooling	Heating II			
	*T_g_* [°C]	*T_m_* [°C]	Δ*H_m_* [J/g]	*T_c_* [°C]	*T_g_* [°C]	*T_m_* [°C]	Δ*H_m_* [J/g]	*X_c_* [%]
PBS	−25.6	121.3	72.39	86.4	−31.7	118.5	70.03	63.49
PBS_15	−32.8	121.9	117.4	83.3	−32.6	104/118.4	75.18	68.2
PBS_30	−32.0	120.8	123.0	84.9	−31.3	105/117.6	93.12	84.4
PBS_45	−28.3	120.1	120.5	84.3	−30.9	104/118.0	96.17	87.2
PBS_60	−34.3	120.0	123.2	84.9	−27.1	104/117.4	98.4	89.1
PBS_90	−37.9	115.7	119.3	82.1	−24.5	98/113.3	104.8	95.0
OP15	−25.1	121.9	71.32	82.1	−33.2	116.7	59.05	62.98
OP15_15	−30.9	124.2	110.5	77.3	−29.1	118.0	76.79	81.9
OP15_30	−30.9	122.5	111.1	77.3	−27.3	118.0	79.5	84.8
OP15_45	−29.8	122.2	118.1	76.8	−28.2	118.5	84.4	90.0
OP15_60	−28.7	122.5	117.9	77.5	−29.4	116.5	86.91	92.7
OP15_90	−30.9	119.4	111.6	75.1	−20.7	116.1	88.2	94.1
OP30	−29.5	119.0	72.15	78.0	−30.2	119.2	51.89	67.21
OP30_15	−26.6	120.7	94.59	76.7	−26.6	116.6	60.65	78.6
OP30_30	−28.2	121.9	98.99	74.2	−25.2	117.8	65.82	85.2
OP30_45	−30.6	120.7	89.35	78.7	−29.9	117.4	69.47	90.0
OP30_60	−28.6	121.3	103.6	74.1	−27.5	119.9	72.4	93.8
OP30_90	−27.4	120.4	83.04	74.2	−28.0	116/119.6	75.34	97.6
WB15	−34.4	118.6	81.5	79.6	−33.6	116.7	69.05	73.65
WB15_15	−34.1	121.9	107.3	83.2	−26.1	117.7	76.39	81.5
WB15_30	−35.1	123.7	111.9	81.4	−30.7	106/121.3	79.78	85.1
WB15_45	−33.5	123.1	108.6	79.9	−26.0	122.4	78.01	88.4
WB15_60	−33.1	118.9	112.7	79.8	−28.7	115.9	94.5	90.2
WB15_90	−25.0	119.6	89.18	75.0	−28.9	118.7	99.18	94.7
WB30	−31.6	118.4	70.0	78.4	−29.5	118.0	61.19	79.25
WB30_15	−35.1	121.2	89.3	81.8	−33.0	105/119.7	62.56	81.0
WB30_30	−29.9	122.0	97.37	80.6	−30.7	105/120.7	70.32	85.0
WB30_45	−32.4	120.7	89.83	78.2	−29.4	120.4	79.04	89.6
WB30_60	−30.3	119.8	84.45	76.7	−17.7	119.4	87.29	87.9
WB30_90	-	117.0	-	76.9	−19.4	116.6	98.92	94.4

**Table 2:** Results of fungi growth assessment tests *.

Batch	Evaluation	PBS	WB15	WB30	OP15	OP30
0	Visual	0	0	0	0	0
S		0	0	0	0	0
I		2	3	4	3	5
I	Microscopic	Not applicable	Not applicable	Not applicable	Not applicable	Not applicable

* Assessment of fungal growth on samples according to the scale: 0—No visible growth under the microscope. 1—Growth invisible to the naked eye, but clearly visible under the microscope, including 1a—covering up to 25%; 1b—up to 50%; 1c—over 50% of the sample surface. 2—Growth visible to the naked eye, covering up to 25% of the tested surface. 3—Growth visible to the naked eye, covering up to 50% of the tested surface. 4—Significant growth, covering more than 50% of the tested surface. 5—Intensive growth covering the entire tested surface. Microscopic assessment required only for batch I, for which the visual assessment showed an increase of 0 or 1.

## Data Availability

The original contributions presented in this study are included in the article. Further inquiries can be directed to the corresponding author.
